# Development of an intervention to support parents receiving treatment in psychiatric inpatient hospital using participatory design methods

**DOI:** 10.3389/fpsyt.2024.1365981

**Published:** 2024-04-02

**Authors:** Abby Dunn, Patrick Fenton, Sam Cartwright-Hatton

**Affiliations:** ^1^Department of Psychology, University of Sussex, Brighton, United Kingdom; ^2^Meadowfield, Sussex Partnership NHS Foundation Trust, Worthing, United Kingdom

**Keywords:** parental mental health, parenting, inpatient units, intervention development, co-production

## Abstract

**Introduction:**

When parents of dependent children are treated in psychiatric inpatient hospital, it typically involves separation of parent and child for the duration of treatment, which can be highly distressing to the dyad and can result in disruption to the parent-child relationship. Parents who have experienced hospitalisation have expressed a desire for their parenting identity to be recognized and appropriately engaged with during their treatment. This recognition includes provision of interventions which support them as parents to limit the impact of their mental health on their children. The current study, the first of its kind known to have taken place, details a collaborative intervention development project for parents receiving inpatient care.

**Methods:**

The current study, the first of its kind known to have taken place, details a collaborative intervention development project for parents receiving inpatient care. This project involved the adaptation and extension of a prior parenting-focused course for parents high in anxiety to meet the needs of parents being treated in inpatient settings. In the first two stages of the three-phase project, patients, carers and mental health practitioners contributed to the revision and delivery plan for the course including developing new content for the intervention. In the final stage, which took the form of a participatory evaluation, the intervention was delivered to 11 parents receiving inpatient treatment who then provided extensive feedback. A series of iterative adaptations to the intervention were made in response to this feedback alongside stakeholder input.

**Results:**

The final intervention comprises five modules focused on exploring the experience of parents alongside specific learning and skills orientated toward boosting their connection with their children during hospitalisation and in readiness for discharge. Preliminary feedback from patients and ward staff has been positive and the process of delivering the project on inpatient wards was associated with no increase in negative clinical outcomes.

**Discussion:**

The successful development of a targeted intervention within inpatient psychiatric units offers a signal that parents treated in this setting welcome the opportunity to be supported in their parenting role. As the first known UK intervention of its kind to be developed in partnership with patients, ward staff and management, it is specifically tailored to the context and needs of this group with the potential to be delivered by a range of health professionals in this setting.

## Background and context

Of the 16,500 psychiatric inpatients in the UK up to 45% may be parents of dependent children (the lack of a clear figure reflects poor data collection regarding parental prevalence) (1). Inpatient treatment requires a temporary breakdown of the family with the likelihood of generating distress to both the unwell parent and their children. There is limited research into the impact of parental hospitalisation for psychiatric needs of parents on child outcomes but there is evidence that it is associated with poorer school outcomes and housing instability (2, 3). Research into the impact of parental serious mental illness (SMI, with which most hospitalised parents are diagnosed) suggests that children of parents with SMI are at high risk of negative mental and physical health outcomes including developing mental health problems of their own (4, 5). Parents with serious mental health difficulties are more likely to be raising children in the context of economic challenges and lone parenthood (6). Furthermore, high levels of readmission (20% within six-months) suggest that the stress and distress of parental hospitalisation is likely to be repeated for many families (7).

Given these risk factors, supporting parents, who are psychiatric inpatients, in maintaining appropriate connection with their children has the potential to benefit both parent and child, and during both treatment and following discharge. Furthermore, parents are clear that they want their parenting role identified and supported when they are receiving psychiatric care (8). Doing so could provide an opportunity to validate a parent’s identity, reduce the stigma associated with parental mental illness and potentially support their recovery. Despite this, no recent intervention supporting this vulnerable group has been identified within the literature (8). Furthermore, a recent survey of mental health services found that mental health workers in inpatient settings were the least likely of all clinical workers to routinely identify parenthood or engage with a patient’s parenting experience or support needs (9). In response to this unmet clinical need, the current study sought to utilise a participatory approach to develop a targeted intervention for parents accessing psychiatric inpatient care.

### Aim of the intervention development programme

The intervention development programme was designed to generate an appropriate and scalable brief intervention for parents who are in receipt of psychiatric inpatient care. It was planned that the intervention would support parents in hospital by a) providing an appropriate space for them to engage with their parenting identity, including the challenges of parenting from hospital and b) provide approaches to strengthen their connection with their child during their hospitalisation and post-discharge. It used participatory methods, through which parents who were currently experiencing hospitalisation contributed to the development of the format and content of the final intervention. It was hoped that collaborators would be empowered through the process of participation at all stages of the project.

In this paper we describe the phased intervention development process and give an overview of the final intervention.

## Methods

### Sample

The following three groups of participants were involved in the intervention development programme:

- adults currently accessing psychiatric inpatient care who were parents of children aged 2-11(to match the target age range of the Raising Confident Children Course),
- National Health Service (NHS) health care professionals (HCP) who work in psychiatric inpatient care,
- partners/carers/supporters of individuals who have accessed psychiatric inpatient care and were parents.

### Inclusion criteria

Parent participants

To maximise access, exclusions were not made on grounds of illness severity. We also included parents who no longer had contact with their children. However, it was required that potential participants had capacity to give informed consent and met the following criteria:

- were aged over 18,
- had been risk-assessed and deemed as having capacity to give informed consent as agreed by clinical team and informed by the Mental Capacity Act 2005 and assessed at the time of involvement and with recognition of potential to change,
- were currently undergoing psychiatric care in an inpatient setting,
- were a parent or carer (including stepparent/foster parent) of a child aged 2-11 (this criterion still applied if child was no longer cared for by the parent),
- had capacity to provide the level of engagement required by the study (this differs for Phase 1, Phase 2, and Phase 3), which was determined through a conversation between the study team and the potential participant.

Staff participants

We sought to involve a wide range of staff members:

- adult (aged over 18),
- currently employed, or acting as a registered volunteer (e.g., MIND Ward Befriending Volunteer), on a psychiatric inpatient ward,
- spent part (a minimum of 25%) of their time in direct contact with patients.

Carers:

- had experience of supporting a parent who has accessed/was accessing psychiatric inpatient care.

### Setting

The intervention was developed within Sussex Partnership NHS Foundation Trust, a large English mental health trust. This trust has 37 inpatient wards. The project was granted ethical approval by West Midlands -South Birmingham Research Ethics Committee [22/WM/0148].

### Recruitment

Participants (parents and staff) were recruited from wards across the trust. The project was designed with a clinical collaborator (PF) who was also a Ward Matron for two adult inpatient wards from which HCPs and patients were recruited. Subsequently, five further wards became involved in the project. The first author (AD) visited wards, attended patient community meetings and multidisciplinary team meetings to promote the project and encourage referral. Carers were recruited via ward activities and AD’s clinical network.

### Development methodology

The project concerns a co-produced adaptation of an existing evidence-based, manualised, parenting intervention (‘Raising Confident Children’/RCC) which was originally designed for use with parents who have anxiety disorders. This adaptation took the form of a ‘pragmatic co-production’ approach, in which the nature of patient and healthcare service engagement was determined by the aim of the project (to develop an intervention drawing upon RCC) and designed to enable these partners in the design process to experience value from the process without requiring them to take on a semi-professional role (10). The design and delivery of the project was also informed by the UK public participation charity INVOLVE’s key principles for co-production: sharing power, including all perspectives and skills, respecting, and valuing the knowledge of all, reciprocity, and building and maintaining relationships (11). The inpatient intervention manual was adapted and extended using relevant theoretical and clinical approaches identified during the development process. This process included drawing on Cognitive Behavioural Therapy, principles of trauma informed practice and an ecological systems framework. However, this was an exploratory and flexible approach, in which the ongoing involvement of end-users and stakeholders shaped both the delivery of the project and the final output. The project had three phases which are described in detail below. For each phase, the process and participants are described alongside key findings which informed subsequent stages.

#### Screening and consent

Participation in phase 1 was defined as patient public involvement (PPI) activity and so formal consent was not required. Participants were paid at the rate determined by the local trust for PPI activities. For phase 2 and phase 3, all participants flowed through the project as follows:

- provided with summary information,
- screened for eligibility,
- provided with information about the study.

Following the provision of informed consent, parents, HCPs, and carers joined the relevant phase of the study.

#### The raising confident children course

The Raising Confident Children course (RCC) provides the foundational components from which a targeted intervention for inpatient parents was developed. As described below, this approach involved both excising material and generating new material suited to the needs of the patient group. The original RCC course was designed by the third author (SCH) and is a two-session, (5 hour) group-based, manualised workshop offered to parents who seek treatment for anxiety within NHS Talking Therapies (mental health primary care) services, where it is delivered by psychological wellbeing practitioners. Its focus is on supporting parents to limit the impact of their anxiety on their children and to promote their child’s confidence. It employs cognitive behavioural and social learning approaches, providing parents with strategies for play, communication, boundary setting and self-management of anxiogenic behaviours. As such, it combines components that are present within many community-delivered parenting interventions, with new components designed for a) parents who experience mental health difficulties and b) parents who specifically experience anxiety. In a randomised controlled trial (RCT), 16% fewer anxiety symptoms were reported in children whose parents were randomised to receive the course, compared to a control group (12)a large (N>1800) national RCT (13, 14) Furthermore, the research team has a track record in effectively adapting the RCC course for other contexts – it has been used by them as the foundation for an intervention designed to support NHS mental health workers who were parents (15).

#### Reflexivity and research group

AD identifies as a white woman and senior lecturer who has conducted extensive research on parental mental health. PF identifies as a white man and is an inpatient unit matron. SCH identifies as a white woman and professor who has conducted extensive research with children and parents with mental health difficulties. All authors have delivered clinical services to adults within the NHS. These roles were acknowledged and held in mind during the delivery of the intervention, analysis and write-up for publication.

### Phase 1: consultation on project parameters

The first author worked with clinical stakeholders and parents to determine the logistics of the project including the duration and frequency of parent evaluation sessions, and to develop an intervention framework for subsequent phases. This consultation took the form of short informal meetings and video calls, all of which were classified as PPI activities.

During this consultation period, the RCC course was dismantled, and the content reduced and re-organised into three sessions. During this process, the elements of the RCC course were arranged into content which would be automatically included for all parents (e.g., play, emotion coaching) and content which would be considered for inclusion only if was found to meet a parent’s personal support needs (e.g., content specifically focused on parental anxiety). This approach reflected the intention that the intervention should be suitable for parents with any mental health presentation (including but not limited to anxiety).

At this stage, the research team also invited input from stakeholders with relevant clinical knowledge, and expertise in other approaches, including trauma-informed practice and social care.

### Participants

One inpatient unit matron (man, 55), one ward manager (woman, 37), and one mental health nurse (woman, 34), two clinical psychologists (woman, 43; woman, 36), one social care professional (woman, 41), one family coach (man, 49) and three parents were involved in these activities (parent demographics in **Table 1**).

**Table 1:** Participant characteristics (parents).

Study phase	Gender	Diagnosis	Time in hospital post consent (weeks)	Youngest child age (years)	Contact with child(ren)	Social care involved
1						
	Female	PTSD EUPD	3	4	Weekly	No
	Female	Bipolar	3	2	Once	Yes
	Female	Depression	16	4	3 visits p/week	Yes
2						
	Female	No diagnosis	4	8	Once	No
	Female	EUPD Depression	2	1	Once	Yes
	Female	Bipolar	2	11	None	None
3						
	Female	Bipolar	8	2	None	Yes
	Female	PTSD Anxiety EUPD	22	4	3 visits p/week	Yes
	Male	Personality Disorder	3	1	Every two weeks	yes
	Female	EUPD Anxiety Depression Eating Disorder OCD	2	6	No	No
	Female	Bipolar	3	4	Once (off ward)	Yes
	Female	EUPD	4	3	None	Yes
	Female	Schizoaffective	3	10	None	No
	Female	Bipolar	8	7 5	None	Yes
	Female	Chronic PTSD EUPD Severe Anxiety Depression	2.5	10	Every two weeks	Yes
	Female	Schizoaffective disorder	2	6	Weekly	No
	Female	ASD ADHD Suicidal Ideation PTSD	36	11	None	No

PTSD, post-traumatic stress disorder; EUPD, emotionally unstable personality disorder; ADHD, attention deficit hyperactivity disorder; ASD, autism spectrum disorder.

### Key findings

There was difficulty in determining the appropriate length and scheduling of sessions because the potential for rapid discharge had to be offset against potential burden of participation. Project design had to reflect the likelihood of parents being discharged before completion of the course and offer ways for them to remain involved. This informed the decision to organise the course content into three sessions.

In addition to the main content, a short (5 minutes) dyadic play-based skill session was included at the close of each session to ensure that each session ended on a playful and positive note, and to provide parents with a new skill to use with their children.

### Phase 2: development of pilot intervention

Interviews were held with parents, HCPs and carers to refine the content of the intervention. These interviews were semi-structured and took place in person either on NHS sites (all parent interviews, of which one was also attended by a clinical psychologist, and HCP interviews), by online video (one HCP), in the community (carers only). The duration ranged from 46 minutes to 118 minutes (M=60.56). These interviews were not recorded (this was in response to feedback in Phase 1) and instead the researcher took written notes which were transcribed into an Excel spreadsheet. Parents and carers were provided with a £10 voucher for taking part. The interviews were structured as follows:

1) The participant was invited to consider the experiences and support needs of parents in hospital.
2) The researcher shared the planned content and structure of the three-session intervention and invited responses regarding its suitability, potential utility, and potential to cause distress.
3) Participants suggested content that should additionally be included in the intervention.
4) Participants reviewed potential supplementary materials (e.g., handouts).
5) Parents and staff were also invited to contribute to the development of ‘distress cards’ for use if a parent wished to silently alert facilitators to their current emotional state (e.g., wished to withdraw from the session).

Participants were invited to feedback by email on the course prior to the start of phase 3. Two HCPs and one carer chose to comment but had no changes to make.

### Participants

Three parents participated, all women, from two wards. One further parent (man) was discharged prior to consent. Four HCPs (one health care assistant with a background in child development, woman, 42; one ward manager, woman 43; two nurses, woman 26, woman, 31) from two wards were interviewed. Two carers also participated: one, man, 41, was the husband of a hospitalised mother. The second, woman, 58, was the mother of a hospitalised parent who cared for her children during her daughter’s treatment.

### Analysis

Phase 2 data were analysed purposively to generate specific suggestions for new content and improvements to materials (course, handouts, distress cards) and processes. Analysis was focused on mapping participant responses to the working version of the course and materials. This process was iterative, so that a suggestion from a participant would be discussed by the research team and then presented to subsequent participants. When a confirmatory response was received, i.e. that a module component was relevant, then it would be retained. Key elements generated through this process are described below. Where content was deemed to be irrelevant or potentially distressing, it would be discussed by the team and with subsequent interviewees. Through this process, a point of consensus was reached when no further content or changes were suggested by participants. At the end of the interview process, the research team compared the responses from participants against the working version of the course to be evaluated in (phase 3).

### Key findings

Participants were positive about the structure and planned content of the new intervention. In particular, the focus on validating the parent was highly valued. Five elements were identified as key for supporting parents in hospital:

1. Support for parents to connect in with their parenting identity “to feel like a mum” (HCP).
2. Support for parents to talk to their children about the experience of hospitalisation and mental health “help parent to help children and carer understand the ward” (carer).
3. Support to manage family visits to reduce child and parent distress “help to manage the time and what to do in it- it’s such a short visit and it always ends really badly” (parent).
4. Support parent and child to connect during hospitalisation “I want to know what to do with them so we can feel like a family” (parent).
5. Helping parents set realistic expectations about their health and their ability to parent their child fully on discharge. “this right now is real life and it may not transform into something completely different” (HCP).

These themes were mapped onto the session delivery plan (**Table 2**) which was then trialled in phase three.

**Table 2:** The content of the three intervention sessions after phases 1 and 2.

*Session 1: You and your child*	
Ice breaker*	Introductions.
Your Parenting experience*	What do you like about being a parent?
Seven confident thoughts*	The 7 ideas we want children to have, to help them feel secure in the world.
Children’s Emotions*	All emotions are OK.
Emotion coaching*	Tuning in to your children’s emotions.
Children’s Sleep*	The importance of good sleep for children.
Play technique^	Fun and relaxing activities to use with your children.
*Session 2: Play and communication*	
Different types of play*	Thinking about the types of play your child needs.
Special play*	A special form of play you can do with your child that makes them feel really close to you.
Helping children feel heard*	Tips for clear communication.
Praise*	How praise boosts children’s confidence
Consequences*	Managing tricky behaviour from children.
Play technique^	Relaxing and fun activities you can use with your children.
*Session 3: Being yourself as a parent*	
Talking to your children^ about your experiences.	Describing your experience in hospital so your child can understand it.
Feelings about going home^	Taking time to understand how you feel about going back to family life.
The myth of the perfect parent*	No parent is perfect, everyone has things that make parenting a bit trickier.
Parenting hotspots*	What things can get in the way of parenting the way you want to, and what we can do to ‘rub the corners off’ them.
Play technique^	Relaxing and fun activities you can use with your children.

Components retained from the RCC course are marked with *. Components marked with ^ were generated within phase 1&2.

### Phase 3: participatory evaluation and refinement

Finally, a process of participatory evaluation was undertaken, through which parents who were in hospital were invited to try out the content of the three-session course in a naturalistic way (intervention) and then after a short refreshment break, to feedback on their experiences of taking part (evaluation).

### Participants

Eleven parents consented to take part in Phase 3 of the project, of whom the majority were women (n=10), white British (n=10, 1=Other), aged 22 to 50 years (M=37.07 SD=9.12) with between 1 and 5 children (M=2). The duration parents had spent in hospital at the point of interview ranged from 2 weeks to 9 months (M=86 SD=112.60) with most having experienced one or more periods in hospital before the present one (n=8). Participant characteristics are presented in **Table 1**.

### Intervention

The intervention took the form of three sessions which were expected to take between 90 minutes and two hours (including a break). The content of the sessions is outlined in **Table 2**. It was co-facilitated by two members of the research team, one of whom is a clinical psychologist (SCH). No members of the ward staff were present during the sessions, but they were provided with an overview of the sessions for inclusion on the participant’s health record and any risk factors arising during sessions were flagged (with the parent’s consent).

### Participatory evaluation

After each intervention session, there was an evaluation session of approximately 30 minutes to one hour. This comprised a discussion designed to support the participant to consider their engagement with the session and its associated materials, and their emotional responses to the content and delivery. It was facilitated using a mapping approach whereby the participant was invited to revisit each of the sections of the session with a series of sticky notes used to collect their comments and impressions. In addition, parents were invited to note down feelings, thoughts, words, comments themselves. The research team also kept a process log during the delivery of the intervention.

Both the intervention and the evaluation sessions were facilitated by the research team. Asking parents to give feedback to the same clinicians who had delivered the intervention can clearly generate bias and inhibit open responding. In order to minimise this risk, the researchers stressed their need to hear an honest view “we really want this to be as good as it can be which means we need to know what is and isn’t working”; the value of the parent as an expert “you are the only person in this room who can experience this as a mum in hospital so you are the expert and we want to hear whatever you have to say” and light-hearted “we have such thick skin, you won’t hurt our feelings.” Parents reported that they were motivated to provide honest feedback in order to improve support for “other mums in this position”.

A planned set of staff workshops, which would have allowed staff input into the process, could not take place due the implementation of a Trust hiring freeze which meant that wards were operating at minimum safe staffing levels and were unable to fund healthcare assistant (HCA) time to enable staff release. Instead, staff were invited to comment on the evolving intervention during team meetings.

### Analysis and revision

**Figure 1:**
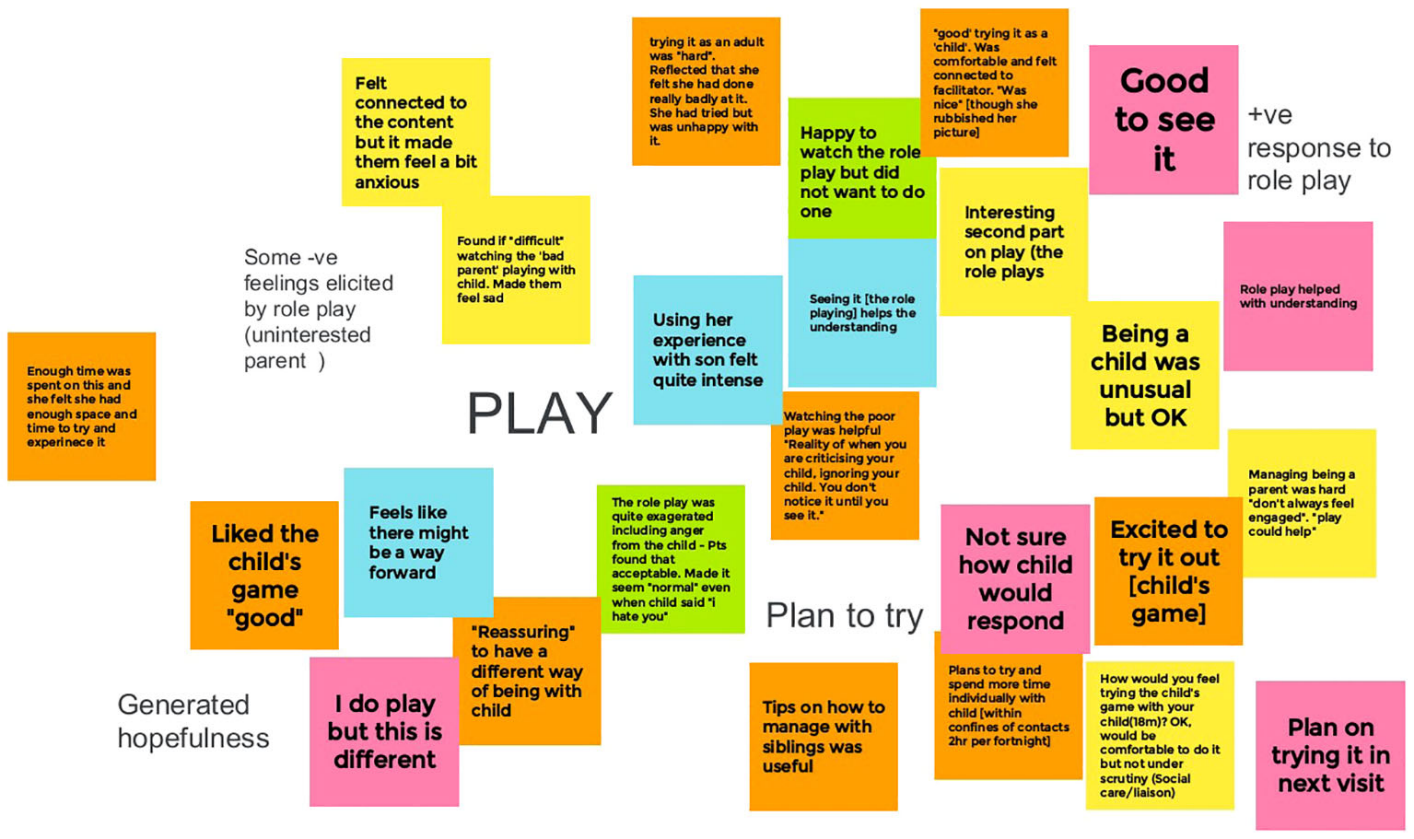
Examples of feedback from five parents on the ‘Play’ component of the intervention.

The intervention development process was iterative, with content, delivery and structural changes made in response to each evaluation session, following each parent’s final session and in response the broader themes that evolved through integrating feedback across parents. Each parent’s comments on the components of each session were mapped on to the feedback of earlier participants using Google Jamboard. Each component had one Jamboard sheet onto which the participant’s notes and responses were assigned a colour and applied (see **Figure 1**). Each Jamboard page was summarised by the research team with the summaries used as a final confirmatory check against each finished module. The facilitators also contributed feedback from their session notes and reflective evaluation.

Ongoing adaptations were made to the intervention throughout this participatory evaluation process which was designed to be flexible and responsive. Ongoing adaption was carried out where there was a clinical need for amendment (e.g., session length caused difficulties in maintaining concentration); feedback indicated that content or an approach was particularly well-received (e.g., role playing the parent’s self-described challenge with their child’s emotions rather than a facilitator-generated example) or poorly received (e.g., negative feedback on a given exercise from multiple parents) AD and SCH discussed these adaptions.

After the participatory evaluation sessions were complete, the draft intervention was compared with the relevant Jamboard pages to ensure it reflected the feedback gathered throughout the process. Each participant note was considered individually and when a specific amendment was proposed it was evaluated against the manual. If it had not been acted upon, this was discussed and resolved by AD and SCH. In revising the intervention, input was also sought from health care professionals to embed good practice with regard to supporting parents in the context of distress, trauma and suicidality. This input included principles of trauma-informed care and practice (e.g., Beckett).

An overview of the final intervention was shared with those parents (n=2) and staff (n=2) who stated they wished to follow-up. The feedback received was positive and primarily focused on satisfaction with the process and the overall aim of the intervention rather than specific changes to content.

### Findings

The process of delivery and of participatory evaluation led to key changes to the format and content of the course. Key areas of course adaptation focused on the following:

1. Exploration. Parents wanted the opportunity to talk about their experiences and their distress at being separated from their children. Parents and facilitators recognised a need to include more time for exploration of family circumstances, the parent’s feelings about their family and the impact of their hospitalisation in the intervention. This included validation of the grief they may be feeling.
2. Opportunity for individual reflection. While parents were willing to engage with the intervention in a small group, they stressed the value of also having some time to reflect on their experiences one-to-one.
3. Focused content. Each session should have one focus, be standalone and include time for exploration and some form of practical tool, activity or approach parents could try with their children during visits or after discharge, and some form of positive ending. This reflected the feedback from parents and sought to mitigate the impact of rapid and unexpected discharge which frequently happened between scheduled sessions.
4. Shorter sessions. Parents did not want sessions to exceed an hour (and for many 50minutes was the maximum duration) as it was identified as hard to concentrate beyond that point.
5. Sessions focused on one topic area. This led to a switch to a five-module intervention.
6. Opportunity to workshop difficult situations. Parents valued the opportunity to bring their specific experiences and challenges into the exercises (for example role playing a challenge that arose during a family visit).

## Results

### Final intervention

**Figure 2:**
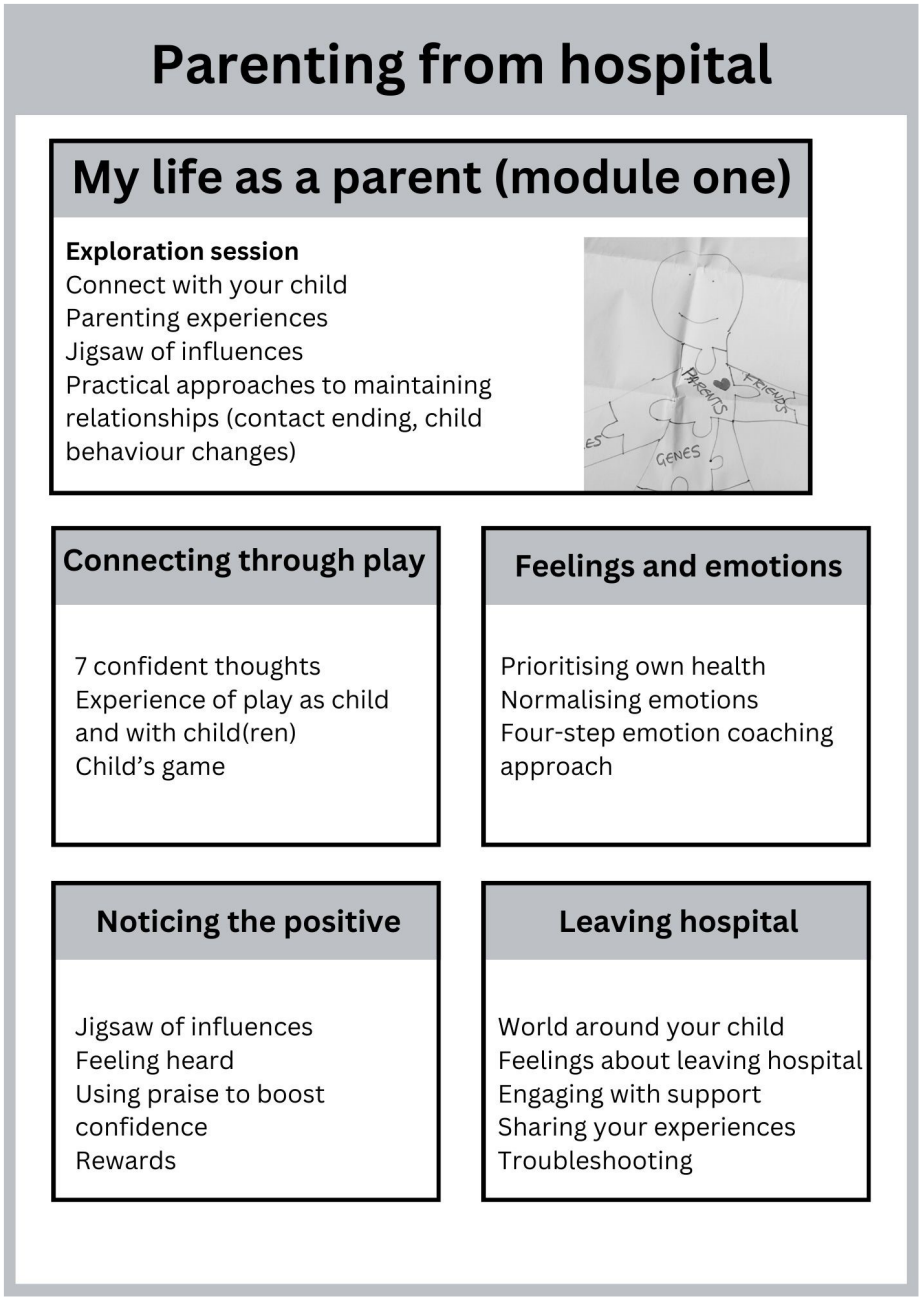
Overview of the final five-session course.

The final intervention (see **Figure 2**) runs across five sessions, which are delivered approximately weekly (but with flexibility to fit the needs of parents and of the ward). Each session begins with a brief introduction including explanation of the ‘distress cards’ and any handouts, and of confidentiality. Each session ends with a quick and fun play technique that parents can use next time they see their child, a check-in regarding the parent’s current mood, a discussion of ward-based support for any feelings that might arise because of the session, and a positive ending statement.

The first session ‘My Life as a Parent’ is designed to be delivered to an individual parent and is focused on giving space to the parent to connect in with their parenting role, and exploration of the experience of parenting from hospital. It offers four optional tools to facilitate the parent’s connection with their child (understanding child’s behaviour, letting your child know you are thinking about them, communication about hospitalisation, how to end hospital visits calmly).

The four remaining sessions are then delivered in whatever order the parent chooses. These sessions can be delivered individually or in a small group. Each of the sessions is organised around a theme (play, emotions, praise and rewards, leaving hospital) and begins with a brief activity focused on connecting with parenting identity followed by an exploration of the theme, specific tools/activities that parents can use to support these ideas (e.g., child-centred play, four-step emotional coaching approach) and an opportunity to experience use of these tools.

### Participant and stakeholder feedback

During and after the intervention design process, the research team kept a log of feedback from ward staff and patients. This feedback has been synthesized as follows:

- Engaging with the intervention was positive for parents: While no formal outcome data was collected from participants, comments from participating parents and staff were positive. In particular, ward staff reported that participants had valued their experience and skills: “She feels much more at ease as a mum and thinks it may help her navigate going home and being present” (ward manager, reporting on a mother who took part).
- The project promoted family-focused practice: Wards that took part in the project reported that the needs of parents were being considered more because of the engagement with the study (e.g., discussion of parenting/parenthood in ward review meetings).
- The process and intervention did not cause heightened distress: Parents who were involved in the study did not experience or report heightened distress and there were no related incidences of self-harm. This was flagged by ward management as an important success and was used to reinforce the value of the work and alleviate the concerns of ward staff who were concerned about potential risks.
- Wards want the intervention to be available: All participating wards have requested future involvement including involvement in a putative feasibility study.

### Dissemination

Three study participants were involved in producing this paper: two parents and one healthcare professional. For reasons of confidentiality, the parent participants did not wish to be named on the final output.

## Discussion

This exploratory project sought to develop an intervention to meet the needs of parents who were receiving treatment in psychiatric inpatient hospital. It was designed to respond to a need identified in the research literature as well as in prior PPI work carried out by the research team. Parents in receipt of psychiatric inpatient care largely describe their experiences in negative terms. Their identity as a parent is often poorly engaged with or supported (8). Children and carers have also identified a need for better provision for hospitalised parents (16, 17). A PPI consultant on the current project, whose wife and mother of two young children was repeatedly hospitalised, described the need as follows: “Being a parent was so important to her, but it was invisible when she was in hospital. There was nothing for us on the outside and nothing for her inside.” Given the lack of identified intervention for this patient group, the project used a pragmatic partnership approach: an extant evidence-based intervention with a track-record of delivery in the NHS was used as a foundation, from which participatory adaptation generated an intervention which parents and ward staff described as being useful and acceptable. Partnership working with the potential end-users of the intervention led to wholesale changes to the structure and content of the intervention.

Inpatient psychiatric wards are frequently operating with unfilled posts and a reliance on bank staff which both contribute to a lack of therapeutic support and a focus on pharmacological stabilisation. However, the growth in trauma-informed practice alongside efforts to embed supportive, therapeutically orientated intervention demonstrate the potential for more holistic care, in which this parenting-orientated intervention could be embedded (18). Research into barriers to family-focused practice in mental health services identify lack of staff skills, confidence, and training, and a lack of any appropriate intervention for parents as inhibitory factors (19). An intervention developed in partnership with end-users, which can be delivered in the inpatient psychiatric context with minimal risk, has potential to enable greater engagement by ward staff. The question remains as to who is most appropriate to deliver this intervention. However, the positive engagement of ward staff in this study (including seeking out of the research team for discussion about the needs of parents), can be interpreted as evidence that the presence of family-focused intervention is a welcome. The current manualised intervention could feasibly be delivered by a range of health care professionals, including occupation therapists and nursing staff. This is advantageous, given their prior knowledge of risk profiles of the parents they would be working with, and it would facilitate ongoing support around parenthood between sessions. Furthermore, as identified by Berry et al., staff in acute settings want to deliver a greater level of holistic care (20). Offering health care staff, the skills to engage with parents can support their development and potentially mitigate risks of burnout. The current study sought to develop an intervention which would be appropriate for use within inpatient units. The clear next step is to evaluate its feasibility drawing upon established evaluative frameworks [e.g., MRC (21)] and taking into account the challenges of implementation within straitened healthcare settings.

### Strengths and limitations

This study was designed to facilitate the engagement of patients using psychiatric inpatients services and successfully involved this group in all stages of intervention design. Furthermore, it involved participants with a range of mental health presentations in the design process and in doing so generated an intervention which can be delivered transdiagnostically, focusing on the commonalities in experience and support needs of parents within the setting. However, most parents recruited into the study were female and White British which is a clear limitation. The bias towards female participants is partly explained by recruitment from a female-only ward but may also reflect biases of ward staff who acted as gatekeepers. The lack of ethnic diversity is reflected broadly in the largely white-British composition of the wards from which parents were recruited. However, this must be purposefully addressed in future activities related to the intervention, to ensure that support does not perpetuate inequalities of access.

While this project was unable to proceed into an evaluation of feasibility or efficacy of the final intervention, it nonetheless provides an important signal that parents in psychiatric inpatient wards have an appetite to engage with a course orientated to their needs as a parent, and that staff are keen to engage parents in this form of intervention. As the first known UK intervention of its kind to be developed in partnership with patients, ward staff and management, it is uniquely placed to offer support which meet the needs of this specific service context.

## Data availability statement

The raw data supporting the conclusions of this article will be made available by the authors, without undue reservation.

## Ethics statement

The studies involving humans were approved by West Midlands - South Birmingham Research Ethics Committee [22/WM/0148]. The studies were conducted in accordance with the local legislation and institutional requirements. The participants provided their written informed consent to participate in this study.

## Author contributions

AD: Conceptualization, Data curation, Funding acquisition, Investigation, Methodology, Project administration, Writing – original draft, Writing – review & editing. PF: Conceptualization, Writing – original draft, Writing – review & editing. SC-H: Conceptualization, Writing – original draft, Writing – review & editing.

## References

[1] Reupert AE Maybery DJ Kowalenko NM. Children whose parents have a mental illness: prevalence, need and treatment. Med J Aust. (2013) 199:S7–9. doi: 10.5694/mja11.11200 25369850

[2] Bell MF Bayliss DM Glauert R Harrison A Ohan JL. Children of parents who have been hospitalised with psychiatric disorders are at risk of poor school readiness. Epidemiol Psychiatr Sci. (2019) 28:508–20. doi: 10.1017/S2045796018000148 PMC6998916 29633682

[3] Konishi A Yoshimura B. Child abuse and neglect by mothers hospitalized for mental disorders. Arch Women’s Ment Health. (2015) 18:833–4. doi: 10.1007/s00737-015-0574-4 26385455

[4] Argent SE Kalebic N Rice F Taylor P. Offspring outcomes when a parent experiences one or more major psychiatric disorder(s): A clinical review. Evidence-Based Ment Health. (2020) 23:113–21. doi: 10.1136/ebmental-2019-300123 PMC10231511 32303570

[5] Pierce M Hope HF Kolade A Gellatly J Osam CS Perchard R. Effects of parental mental illness on children’s physical health: Systematic review and meta-analysis. Br J Psychiatry. (2020) 217:354–63. doi: 10.1192/bjp.2019.216 31610824

[6] Alegría M Ludman E Kafali EN Lapatin S Vila D Shrout PE. Effectiveness of the engagement and counseling for Latinos (ECLA) intervention in low-income Latinos. Med Care. (2014) 52:989–97. doi: 10.1097/MLR.0000000000000232 PMC4201237 25310525

[7] Osborn DPJ Favarato G Lamb D Harper T Johnson S Lloyd-Evans B. Readmission after discharge from acute mental healthcare among 231 988 people in England: cohort study exploring predictors of readmission including availability of acute day units in local areas. BJPsych Open. (2021) 7:e136. doi: 10.1192/bjo.2021.961 34275509 PMC8329766

[8] Dunn A Christiansen H Elsby-Pearson C Kramer J Swinburn E Platt B. Psychiatric in-patients who are parents: what interventions are tailored to their needs and how do they experience care? A systematic review and data synthesis. BJPsych Open. (2023) 9:e111. doi: 10.1192/bjo.2023.67 37345520 PMC10305023

[9] Dunn A Startup H Cartwright-Hatton S. Adult mental health service engagement with patients who are parents: Evidence from 15 English Mental Health Trusts. Br J Clin Psychol. (2021) 61:335–48. doi: 10.1111/bjc.12330 34609005

[10] Kaehne A Beacham A Feather J. Co-production in integrated health and social care programmes: A pragmatic model. J Integrated Care. (2018) 26:87–96. doi: 10.1108/JICA-11-2017-0044

[11] NIHR. Guidance on co-producing a research project (2021). Available at: https://www.learningforinvolvement.org.uk/wp-content/uploads/2021/04/NIHR-Guidance-on-co-producing-a-research-project-April-2021.pdf.

[12] Cartwright-Hatton S Ewing D Dash S Hughes Z Thompson EJ Hazell CM. Preventing family transmission of anxiety: Feasibility RCT of a brief intervention for parents. Br J Clin Psychol. (2018) 57(3):351–66. doi: 10.1111/bjc.1217 29575043

[13] Palmer E Woolgar M Carter B Cartwright-Hatton S Challacombe FL. Preventing anxiety in the children of anxious parents – feasibility of a brief, online, group intervention for parents of one- to three-year-olds. Child Adolesc Ment Health. (2023) 28:33–41. doi: 10.1111/camh.12596 35983606

[14] Dunn A Alvarez J Arbon A Bremner S Elsby-Pearson C Emsley R. Effectiveness of a web-based intervention to prevent anxiety in the children of parents with anxiety: protocol for a randomized controlled trial. JMIR Res Protoc. (2022) 11:e40707. doi: 10.2196/40707 36355406 PMC9693706

[15] Dunn A Dixon C Thomson A Cartwright-Hatton S. Workplace support for mental health workers who are parents: A feasibility study. Front Psychol. (2022) 0:2806. doi: 10.3389/fpsyg.2022.854065 PMC9262046 35814147

[16] Scholes A Price O Berry K. Women service users’ experiences of inpatient mental health services and staff experiences of providing care to women within inpatient mental health services: A systematic review of qualitative evidence. Int J Nurs Stud. (2021) 118:103920. doi: 10.1016/j.ijnurstu.2021.103920 33857788

[17] Källquist A Salzmann-Erikson M. Experiences of having a parent with serious mental illness: an interpretive meta-synthesis of qualitative literature. J Child Fam Stud. (2019) 28:2056–68. doi: 10.1007/s10826-019-01438-0

[18] Berry K Raphael J Wilson H Bucci S Drake RJ Edge D. A cluster randomised controlled trial of a ward-based intervention to improve access to psychologically-informed care and psychological therapy for mental health in-patients. BMC Psychiatry. (2022) 22:1–15. doi: 10.1186/s12888-022-03696-7 35114980 PMC8815159

[19] Gregg L Adderley H Calam R Wittkowski A. The implementation of family-focused practice in adult mental health services: A systematic review exploring the influence of practitioner and workplace factors. Int J Ment Health Nurs. (2021) 30:885–906. doi: 10.1111/inm.12837 33792149

[20] Berry K Raphael J Haddock G Bucci S Price O Lovell K. Exploring how to improve access to psychological therapies on acute mental health wards from the perspectives of patients, families and mental health staff: qualitative study. BJPsych Open. (2022) 8:e112. doi: 10.1192/bjo.2022.513 35698827 PMC9230441

[21] Skivington K Matthews L Simpson SA Craig P Baird J Blazeby JM. A new framework for developing and evaluating complex interventions: update of Medical Research Council guidance. (2021) 374:n2061. doi: 10.1136/bmj.n2061 PMC8482308 34593508

